# Supplementary material for: Are carbon emissions trading and green financial instruments synergistic? -Comprehensive quantitative research based on content analysis
Source: PLoS One. 2024 Mar 7;19(3):e0298601. doi: 10.1371/journal.pone.0298601 (PMC10919601; doi:10.1371/journal.pone.0298601)
Supplement: S1 Data — (ZIP) [file pone.0298601.s001.zip › Data/地级市Z_F工作报告.zip!/┴╔─■/╠·┴δ╩╨/└┤╫╘╣1⁄2╓┌║┼í╛┬φ┐╦╩2╛▌═°í┐_2007.docx]

各位代表：

　　现在，我代表市人民政府向大会报告工作，请予审议，并请市政协委员和其他列席会议的同志提出意见。

　　一、2006年工作回顾

　　过去的一年，我们在省委、省政府和市委的正确领导下，在市人大依法监督、市政协民主监督与大力支持下，团结和带领全市各族人民，坚持以邓小平理论和“三个代表”重要思想为指导，以科学发展观统领全局，统筹兼顾，突出重点，勠力同心，攻坚克难，全面完成了市五届人大三次会议确定的各项任务目标，国民经济和社会事业取得了显著成就，多项工作实现了重大突破，创造了铁岭发展史上的奇迹。

　　——国民经济实现了又好又快的发展。初步统计，全市地区生产总值实现323．7亿元，增长20．1％，其中，一二三产业增加值实现79．7亿元、139．1亿元牙104．9亿元，分别增长11．2％、27．2％和1 8．5％。万元生产总值综合能耗下降3％。地区财政收入32．81亿元，增长2 3．6％。地方财政一般预算收入16．28亿元，增长27．3％。一般预算支出44．17亿元，增长25％。规模以上工业增加值实现119．7亿元，增长32．8％。全社会固定资产投资完成202．8亿元，增长89．6％。社会消费品零售额111．5亿元，增长15％。实际利用外资3200万美元，增长104％。外贸出口1亿美元，增长23％。金融机构存款余额334．6亿元，贷款余额268．7亿元，分别比年初增长10．7％和14％。城镇居民人均可支配收入7437元，增长13．9％。农民人均纯收入4222元，增长12．4％。

　　——沈铁工业走廊呈现崭新形象。102线和铁三线双向八车道拓宽改造工程竣工通车。17个重点工业园区建设取得了突破性进展，40平方公里起步区实现了“六通一平”。园区对外吸引力大大增强，当年入驻企业273家，固定资产投资80亿元。沈铁工业走廊正朝着“工业集中、产业集聚、土地集约、功能完善、管理科学、环境优美、生态良好”的新型工业基地方向发展，被写入省第十次党代会报告，这是我市经济建设最辉煌的业绩，在铁岭经济发展史上具有里程碑意义。

　　——项目年活动和招商引资实现了重大突破。共楷引进域外投资项目726个，实际到位资金159亿元，其中亿元以上项目65个。铁岭电厂二期、清河电厂以大代小、开原30万吨燃料乙醇等一批重大项目开工建设，昌图辽能协鑫风力发电实现了当年签约、当年建设、当年投产。煤矸石发电、秸秆发电、亮中煤田、煤化工等项目取得了重要进展。总投资5 0亿元的东北国际物流中心项目已经签约，高尔夫球场投入使用，东北土特产品交易中心基本建成，果蔬批发市场项目进展顺利。先后引进了北京京润蓝筹公司、中粮集团、深圳华南城集团等一批国内外知名企业来我市投资兴业。争取省技改贴息5 2 0 0万元，实施技改项目1 7 9项，拉动固定资产投资5 5亿元。通过项目年活动和招商引资，促进了两大基地加速发展，促进了五大主导产业初步形成，促进了一批骨干企业做大做强。

　　——中心城区建设实现了历史性的跨越。新城区建设一年打基础的目标如期实现。按照建设适宜人居、适宜创业、适宜休闲度假的中国北方水城的目标，高水平地完成了新城区的规划。起步区基础设施全面开工建设。凡河新村建成并交付使用。莲花湖湿地恢复建设工程列入国家规划，凡河整治、凤冠山、如意湖、天水河工程正在紧张施工。垃圾处理厂一期等1 2项改造提升银州区功能的重点工程全部完成，被评为省级园林城市。积极推进5个副中心城市建设，西丰、开原、调兵山城市建设和管理迈上新台阶，城市面貌焕然一新。20个中心集镇规划和建设工作已经起涉。

　　——社会主义新农村建设形势喜人。县域经济发展势头强劲，7个县(市)区地区生产总值、地方财政一般预算收入、工业增加值等主要指标平均增幅达到30％以上。粮食喜获大丰收，总产量66．28亿斤，创历史最好水平。农业经济结构不断调整优化。新建村外畜牧小区60个，畜牧业产值占农业总产值的比重达到47％；新开发蔬菜保护地11．9万亩，实现蔬菜产业产值19亿元，增长17．8％；实现林业产值11．3亿元，增长25％；绿色和无公害农产品生产面积达到223万亩，我市被确定为全国绿色农业示范区。农业产业化迈出新步伐。新建固定资产投资2000万元以上产业化项目34个，农业产业化实现总产值211亿元，增长20．6％。积极推广昌图县宝力镇有组织劳务输出的经验，培训农民3．5万人次，劳务输出5．16万人，促进了农民增收。农业基础设施进一步改善，完成骨干工程322项，人畜饮水工程46处，治理水土流失面积16万亩，植树造林28．2万亩，有望连续夺得省“大禹杯”。农业机械化水平有新提高，农机总动力比上年增加8万千瓦。农村公路网建设得到加强，又有112个村通上了油路。社会主义新农村建设试点工作全面展开。农村新能源建设成效显著。广泛开展农村爱国卫生运动，村容村貌发生了较大改观。

　　——民生工程取得明显成效。筹集资金4．26亿元，新改造集中连片棚户区44．4万平方米，动迁居民全部按时回迁。筹集资金1．52亿元，安置实名制就业96628人，有就业愿望的零就业家庭至少实现1人就业。筹集资金7．8亿元，确保了企业离退休人员养老金和享受失业保险待遇人员失业金按时足额发放。筹集资金9729万元，使全市城乡15．2万名低保对象实现了应保尽保并提高了保障标准。筹集资金1506万元，改造农村低保户、优抚户D级危房1004户，已全部完工并交付使用。筹集整村推进扶贫开发资金1540万元，落实项目77个，受益人口4．3万人。筹集资金1000万元，建设市区36处社区办公用房，银州区社区办公用房全部得到解决。筹集资金2700万元，广泛开展社会救助，困难群体和灾民的生产生活得到妥善安置。筹集资金4680万元，新建和扩建基层派出所办公用房106处、警务室478处，被公安部确定为全国公安“三基”工程建设示范市。

　　——和谐铁岭建设成绩斐然。科技工作得到加强，建立企业研发中心4家，高新技术企业发展到2 7户，培养农村科技经纪人1 1 5 2人。筹集资金1．8亿元，新建九年一贯制学校1 1所，改扩建农村中学9所，改造农村中小学D级危房6．3万平方米。筹集资金9200万元，在全省率先建成远程教育平台，促进了教育事业均衡发展。职业教育资源得到整合，铁岭师专与美国尔班纳大学和班尼迪克大学合作办学，实现了高等教育的新跨越。文化事业日益繁荣。在全国第六届工笔画大展中再创佳绩，“铁岭工笔画，在美术界的影响进一步扩大。铁岭二人转、铁岭指画分别列入国家和省第一批非物质文化遗产名录。“铁岭明月圆．中华一家亲，海峡两岸各民族中秋联欢活动在我市成功举办，成为文艺和对台交流的一大盛事。筹集资金5541万元，推行新型农村合作医疗制度，参合率达到88．92％，参合农民受益面达到30．9％。农村卫生院建设和经营管理体制改革成效明显。城市社区卫生服务机构达到77个，覆盖面达到85％。对城乡困难群众实施医疗救助，缓解了看病难问题。体育运动蓬勃开展，在全省率先实施“百万农民健身工程”，在省十运会比赛中获奖牌72枚，其中金牌28枚。完成80个20户以上村屯广播电视村村通工程，广播电视节目制作播出实现了数字化。加大出生人口性别比升高问题综合治理力度，继续稳定了低生育水平。广泛开展社会主义荣辱观教育，不断深化城乡共建活动，被评为省级创建文明城市工作先进市。理顺了应急管理体制，处置突发事件的能力进一步增强。切实加强信访和市民投诉工作，解决了大量事关群众切身利益的实际问题。严格执行安全准入制度，加大安全监管和事故查处力度，安全生产形势平稳。平安铁岭创建活动扎实有效，刑事犯罪、治安犯罪和经济犯罪呈全面下降趋势，成为全省最稳定地区之一，有望获得全省社会治安综合治理工作先进市称号。

　　——体制机制创新取得明显进展。国有粮食企业产权制度改革成效显著，被评为“全国国有粮食企业改革工作先进单位”。集体林权制度改革取得突破性进展，昌图县平原区林权改革创造了经验，得到国家林业局的充分肯定。国有企业改革进展顺利，列入省计划的1 26户企业改制任务全部完成，省划归我市的国有企业改革工作稳步推进。完成产权交易45项，成交额1亿元。成功回购信达和长城公司债权17．4亿元，为154户企业解开了主债务链。加大了国有土地使用权招拍挂的力度，收益突破10亿元。积极推进事业单位人事制度改革，市直事业单位全面推行了人员聘用制。市商业银行已获国家银监会批准筹建。

　　——经济社会发展环境进一步优化。深化行政审批制度改革，行政审批项目进中心率达到93％。实行了“限时办结、超时默认”制度，办事效率进一步提高。认真办理人大代表建议131件、政协提案423件，办结率、见面率100％，满意率98％，对加强和改进政府工作起到了促进作用。制度建设上了一个新水平，被确定为“全国市级规范性文件备案审查工作示范单位”。积极推进行政执法体制改革，交叉执法、多头执法问题得到有效解决。严格落实行政执法责任制和过错追究制，强化了法制监督。成立中小企业法律顾问团，维护了中小企业的合法权益。加大政务公开工作力度，保障了人民群众的知情权、参与权和监督权。开展选优评差竞赛活动，提高了各部门加强软环境建设的自觉性。切实加强廉政建设，各级领导干部廉洁自律意识进一步增强。在全省率先开展公务员高层次、正规化培训，公务员队伍素质不断提高。

　　外事、侨务、台办、旅游、物价、统计、审计、民族、宗教、综合执法、档案、工商、药品监督、质量技术监督、出入境检验检疫、邮政、通信、供销、烟草、房产、气象、人防、国家安全、司法、消防、新闻出版、地震、接待、残联、商业保险、驻外联络、仲裁、市志、老龄等工作也都取得了新成绩。

　　各位代表，一年来的成就令人欣慰，全市上下呈现出政通人和、百业俱兴、众志成城、心齐气盛的喜人局面。更重要的是找到了抓纲带目、合力攻坚、典型引路、狠抓落实的有效方法：显示出攀高比强、敢想敢干、不甘人后、奋勇争先的弘大气魄；体现了顾全大局、不计得失、顽强拼搏、忘我奉献的可贵精神；形成了说干就干、雷厉风行、咬住重点、攻坚克难的过硬作风。这是一年来取得各项成就的根本原因，也是做好今后各项工作的强大动力。在此，我代表市人民政府，向辛勤工作在各条战线上的全市各族人民，向大力支持政府工作的各民主党派、工商联、无党派人士、人民团体以及社会各界人士，向解放军驻铁部队指战员和武警官兵、中省直驻铁单位，向广大域外投资客商，向所有关心支持铁岭改革开放和现代化建设的海内外朋友表示衷心的感谢并致以崇高的敬意！

　　在看到成绩的同时，我们也清醒地看到，全市经济社会发展还存在一些深层次的矛盾和问题：一是经济总量小、工业化水平低、城镇化水平低的局面还没有得到根本扭转。二是当前人民群众的生产和生活还面临着许多亟待解决的困难和问题。城镇居民人均可支配收入与全省平均水平差距很大；城市失业人员和农村富余劳动力数量众多，就业形势比较严峻；因病致贫、因病返贫、困难群众看病难、看病贵问题仍然比较突出；城乡弱势群体的救济工作仍需要加强。三是影响发展的体制性、机制性障碍还比较多，深化改革的任务还很艰巨。四是“三农”问题比较突出，农民持续增收难度大，农村各项社会事业发展严重滞后。五是政府职能转变还不适应新形势、新任务的要求，少数部门本位主义、小团体利益思想比较严重，执行力、公信力和依法行政能力还有一定差距，经济社会发展环境有待进一步改善。这些问题已经弓l起我们的高度重视，将在改革和发展过程中认真加以解决。

　　二、2007年的主要工作

　　2007年，政府工作的指导思想是：以邓小平理论和“三个代表”重要思想为指导，认真贯彻落实党的十六届六中全会和中央经济工作会议精神，以科学发展观为统领，按照省第十次党代会部署，着力调整经济结构和转变增长方式，着力加强资源节约和环境保护，着力推进改革开放和自主创新，着力促进社会发展和解决民生问题，深入实施五大战略，全力推进工业化和城镇化，加快社会主义新农村建设步伐，大力发展以现代物流为重点的现代服务业，努力构建和谐铁岭，实现沈铁工业走廊建设、县域经济发展、新城区建设、现代物流园区建设的新跨越，把建设新铁岭大铁岭的宏伟事业推向一个新阶段，以优异成绩迎接党的十七大胜利召开。

　　主要预期目标是：地区生产总值390亿元，增长20％。地区财政收入40亿元，增长22．5％。地方财政一般预算收入20亿元，增长22．8％。万元生产总值能耗下降4％。主要污染物排放总量减少2％。城镇登记失业率控制在5％以内。规模以上工业增加值165亿元，增长33％。全社会固定资产投资260亿元，增长3 0％。社会消费品零售额127亿元，增长14％。城镇居民人均可支配收入8180元，增长10％。农民人均纯收入4560元，增长8％。县(市)区地区生产总值、地方财政一般预算收入和规模以上工业增加值增长30％以上。

　　为实现上述目标，将着力抓好以下10项重点工作：

　　(一)以沈铁工业走廊建设为中心，加速推进工业化

　　建设沈铁工业走廊，是抓住东北老工业基地振兴机遇的重要着力点，是进一步扩大对外开放的重要突破口，是建设新型产业基地的重要载体，意义非常重大。一是把沈铁工业走廊建设成为科技含量高、资源消耗低、经济效益好、环境污染少的新型工业化示范基地。今年要在推进项目建设上实现新突破，通过引资建设一批重大项目，促进五大主导产业尽快形成，促进一批骨干企业做大做强，带动产业结构优化升级。二是把工业园区建设作为重点，通过园区建设发展产业集群，提高产业集聚度，推动工业向规模化、集约化方向发展。今年要在基础设施建设上实现新突破，完善园区功能，提高园区的吸引力和项111承载力。三是把沈铁工业走廊建设成为对外开放的窗起1：1，建设成为全市开放程度最高、政策最优惠、功能最齐全的经济“特区”，带动全市经济在扩大对外开放中实现快速发展。今年要在招商引资上实现新突破，面向国内外，积极吸引国有、民营、境外资本到各类工业园区投资上项目。四是把深化改革放在突出位置，实现体制机制创新，为加速推进工业化创造良好的制度环境和政策环境。今年要着力在加快行政审批制度改革、提高行政效率上下功夫，着力在完善公共服务政策体系、提高公共服务质量上下功夫。建立重点工业园区管委会，对园区实行封闭式管理，充分履行规划、建设、招商、管理、服务职能。五是充分发挥科技在推进工业化进程中的支撑和引领作用，将沈铁工业走廊打造成科技创新走廊。今年要加快用高新技术和先进适用技术改造传统产业，使全市技术改造工作实现重大突破，特别是支持那些主业突出、竞争力强的企业进行“二次创业”。加快建立以企业为主体、以市场为导向、产学研相结合的技术创新体系，努力实现科技工作提速、主要指标翻番的目标。充分发挥高新技术园区作用，着力抓好日东科技园和环保产业园建设。努力把铁岭高新技术产业开发区和铁岭经济开发区建设成为全市改革开放先导区、新型工业化示范区，成为沈铁工业走廊的两面旗帜。

　　(二)加快发展县域经济，全面推进新农村建设

　　以推动农村经济发展为中心任务，以增加农民收入为目标，以发展壮大县域经济为重要载体，全面推进社会主义新农村建设，努力实现农民增收、农业发展、农村和谐。一是强化工业强县理念，大力调整县域经济结构。依据比较优势，确定县域经济主导产业，以抓好工业园区建设为载体，努力提高县域工业的集聚度，扩大县域经济总量，壮大县级财力，逐步建立以城带乡、以工促农的长效机制。用工业理念谋划农业，以建设全国优质特色农产品生产加工基地为目标，积极推进农产品标准化生产和产业化经营。重点是做大做强20个产业化龙头企业，打造20个优质农产品品牌，新上固定资产投资2000万元以上龙头企业20家。二是加快构建现代农业产业体系。继续实施“畜牧业倍增计划”，新建畜牧小区70个，提高畜牧业规模化、集约化水平。强化动物防疫工作，确保动物产品安全。推进25个种植业优势产区建设，加快买施“四个100万工程”，力争到“十一五”末期，蔬菜、优质水稻、榛子和花生种植面积分别达到100万亩。大力发展绿色和无公害农产品，努力提高农产品质量。全面完成集体林产权制度改革，释放林业经济活力，完成植树造林20万亩，实现林业产值14亿元，增长25％。三是积极推广现代经营形式。大力发展各种形式的农村专业合作经济组织和民间行业协会，提高农民的组织化程度，健全社会化服务体系。四是加快培养新型农民。继续推广昌图县宝力镇经验，着力抓好有组织的劳务输出，培训农民3．5万人，新增劳务输出5万人。五是加强农村基础设施建设。新建农村公路500公里，建设开原老城、西丰平岗、调兵山大明3个客运站，开工建设西辽铁路。重点抓好150项骨干工程，新增、改善灌溉面积8万亩，治理水土流失面积10万亩。全力支持西丰诚信水库建设，年内实现大坝合龙蓄水。完成饮水工程305处，解决17万人安全饮水问题。提高农业装备水平，农机总动力达到178万千瓦。六是改善农民的生产生活环境。新建秸秆气化工程15个，大中型沼气池4个，“四位一体”生态模式1060个，全面整治村容村貌。

　　(三)继续做大做强中心城市，加快城镇化进程

　　全面加快新城区建设步伐。继续实施凡河治理、凤冠山堆筑、莲花湖湿地恢复、天水河、如意湖、园林绿化等工程，全力打造中国北方水城。以道路、给排水、供热、供气、供电、污水处理等为重点，完成5平方公里起步区基础设施建设。开工建设市县行政中心、居民住宅、技师学院等一批重点项目，实现两年出形象的目标。

　　以“五城联创”为载体，进一步提升城市建设和管理水平。“十一五”期间，要争创国家卫生城市、国家园林城市、国家环保模范城、全国文明城市和全国双拥模范城，2008年要取得阶段性成果。今年，要围绕“五城联创”的基础设施建设和解决人民群众最关心、最直接、最现实的问题，在继续抓好客运站和铁岭大剧院工程的同时，重点抓好10个方面的实事。1．改造广裕街。2．改造西出口。3．对城乡结合部环境进行综合整治。4．对小街小巷及社区环境进行综合整治。5．改造完善南马路商业街。6．进一步搞好城市绿化。7．以物业管理、清理城区自备井和拆除违章建筑等为重点，切实加强城市管理。8．改造5万平方米以下棚户区。9．建设新的热源厂。10．开通铁岭外环公交线路。搞好市直和铁岭县、银州区行政事业单位资产整合，实现资产的合理配置。

　　加快副中心城市建设。高起点搞好副中心城市规划修编，打造特色城市。创新城建体制机制，拓宽融资渠道，加快城市基础设施建设步伐，提升城市功能。采取有力措施，积极推进中心集镇建设。

　　(四)以现代物流业为重点，大力发展金融、信息、物流、大力发展现代服务业咨询、中介、文化、商贸、旅游等现代服务业。把现代服务业作为一个支柱产业来抓，是今后一个时期促进经济结构调整和产业优化升级、促进中心城市功能完善的重要内容。依托沈阳，服务东北，以发展现代物流业为突破口，以建设东北国际物流中心为标志，努力把铁岭建设成为东北地区现代物流基地，促进现代服务业全面发展。今年的工作重点：一是全力支持华南城集团投资50亿元建设东北国际物流中心，成立组织，创造条件，加强协调，确保上半年全面开工建设。二是引进和培育一批现代物流企业，实现物流经营的现代化、专业化和规模化。三是加快金山国际广场等在建项目的建设进度，使其早日建成并投入运营。四是深入开展“万村千乡市场工程”，积极发展农村物流，大力开拓农村市场。五是搞好旅游业规划和建设，整合、开发城乡旅游资源，加快发展旅游产业。六是编制好现代服务业发展规划，降低准入门槛，制定扶持政策，培养和引进专业人才，为发展现代服务业创造条件。

　　(五)深入开展项目年活动，实现招商引资规模、质量和效益的新突破

　　继续分解落实招商任务，对在招商引资中做出突出贡献的单位和个人予以重奖。实行各级领导包扶点项目责任制，加强对重点项目进展情况的协调和调度，及时解决项目运作过程中的各种实际问题。切实加强项目源建设，精心开发、包装、储备一批项目，为项目年活动提供载体。用好用足上级政策，继续加大向上争取项目和资金的力度。围绕资源优势，立足产业基础，集中精力抓好工业、能源交通、农业产业化、城镇基础设施建设以及第三产业项目。境内以上海、深圳、东莞、温州、泉州等地为重点，境外以香港、台湾、欧洲、北美、韩国、日本、东南亚等地区为重点，精心组织招商活动。创新招商方式，提高招商质量，务求招商实效。今年实际到位资金要达到200亿元，增长30％；实际利用外资6400万美元，翻一番。积极培育新的出口增长点，外贸出口实现1．2亿美元，增长20％。

　　(六)做好财税金融工作，提高经济社会发展的资金保障能力

　　大力组织税收收入，强化税源管理，推进税收信息化建设，规范税收减免行为，做到应收尽收，努力做大财政“蛋糕”。牢固树立过紧日子思想，强化预算约束，严格预算执行，大力压缩会议费、考察费、车辆运行费、招待费，做到开源与节流并重。健全公共财政体制，优化财政支出结构，重点向农业、教育、卫生、文化、科技、就业再就业、社会保障、社会救助等领域倾斜。深化部门预算改革，完善国库集中收付制度。充分发挥筹融资平台作用，加大筹融资力度，促进经济和社会发展。建立健全绩效考核机制，加强对政府投资项目的监管。推行审计公开制度，严肃财经纪律。进一步推动金融生态环境建设，以培植信用企业为重点，积极打造“诚信铁岭”，增强区域融资的吸引力。加强银企合作，努力增加信贷投放，加大对地方经济发展的支持力度。大力清收金融机构不良贷款，积极防范和化解金融风险。

　　(七)以解决民生问题为重点，努力构建和谐铁岭

　　一是认真抓好就业再就业。特别要解决好贫困群体、下岗失业人员、失地农民和大中专毕业生就业。培训下岗失业人员2万人，实现再就业5万人，确保零就业家庭至少有1人稳定就业。二是进一步完善社会保障体系。加快“五险合一”社保网络建设，加强农民工和失地农民的社会保障工作，建立健全农民工工资支付保障制度，维护其合法权益。进一步完善社会救助体系，落实城乡低保制度，切实解决困难群体面临的各种实际困难，逐步建立社会保险、社会救助、社会福利、慈善事业相衔接的覆盖城乡居民的社会保障体系。研究探索扶贫工作的新机制，加快推进整村扶贫开发。及时开展救灾救济，确保灾民的生产生活。三是积极化解各种社会矛盾。健全社会舆情汇集和分析制度，加强信访、投诉、仲裁和人民调节工作，形成有效化解社会矛盾纠纷的长效机制。进一步理顺社区工作关系，加强社区工作者队伍建设，充分发挥社区在服务居民、化解矛盾、维护社会稳定等方面的作用。加强宗教管理，积极引导宗教与社会主义和谐社会相适应。进一步理顺应急管理体制，完善突发事件监测预警机制，建立应急信息平台和指挥平台，妥善处置各种突发事件。四是深入开展平安铁岭创建活动。不断强化社会治安防控体系，把社会治安综合治理的各项措施落实到基层。依法严厉打击严重刑事犯罪和重大经济犯罪，增强人民群众的安全感，争创全省最稳定地区。落实企业安全生产主体责任，认真整治安全隐患，加大安全生产违法行为处罚力度，严格落实“三同时”制度。坚决遏制重特大安全生产事故、有影响的群体性事件、重大刑事犯罪案件的发生。切实加强环境保护，把节约能源资源放在更加突出的战略位置，努力实现人与自然的和谐。

　　(八)加快发展各项社会事业，让人民群众共享改革和发展成果

　　一是开展“教育年”活动。重点实施9大工程，即农村中小学D级危房改造和九年一贯制学校建设工程、新区教育园区建设工程、义务教育“两类新三片”规划达标工程、职业教育质量提升工程、和谐校园建设工程、现代远程教育工程、教师素质提升工程、捐资助教工程、高等教育对外合作与交流工程。二是加强以农村为重点的卫生工作。建立以县级医院为龙头、乡镇卫生院为前沿、村卫生所为网底的三级医疗预防保健网络，进一步完善新型农村合作医疗制度。加强医护人员培训，实行城乡对口帮扶。建立稳定的农村卫生事业经费保障机制，逐步解决农民看病难、看病贵、看病差的问题。继续加强城市社区卫生工作，形成政府主导、部门协作、社会参与的社区卫生发展支持体系，调整优化社区卫生资源布局，改善社区卫生服务机构的基础设施和设备条件，为城市居民提供方便、快捷、优质、价廉的卫生综合服务。加强食品药品监管，确保群众消费安全。三是认真抓好文化、计划生育、广播电视、体育、人才等工作。以社会主义核心价值体系为根本，扎实推进和谐文化建设。深入开展以社会主义荣辱观为核心内容的思想道德建设，广泛开展和谐社区、和谐乡村建设和城乡共建活动，完善公共文化服务体系，加强基层文化建设，提高文艺创作水平，打造一批文艺精品，大力发展文化产业。综合运用法律、行政、教育、经济等手段，不断提高计划生育管理和服务水平，建立健全长效工作机制，稳定低生育水平。完成200个20户以上村屯广播电视村村通工程，启动银州区和新城区有线数字化广播电视工程。开展以“与奥运同行”为主题的群众性体育活动，继续推进“百万农民健身工程”，建成80个农村体育广场，办好市第六届全民运动会。实施人才环境优化、人才素质提升和人才引进“三大工程”，为经济建设和社会发展提供人才和智力支持。

　　(九)努力实现体制机制创新，为经济社会发展提供强大动力

　　打好国有企业改革攻坚战。完成省划归我市管理的精工等企业的改制重组工作，做好移交开原市管理的市属企业改制工作，推进已改制企业完善法人治理机构，建立现代企业制度。依法履行出资人职责，加强国有资产监管，强化资本运营。加强对农村税费改革资金的管理，指导国有农场实施税费改革。全面完成集体林产权制度改革任务。深化粮食流通体制改革，确保粮食安全。全面实施公务员法，完成事业单位人事制度改革，积极推进事业单位机构改革。

　　毫不动摇地支持非公有制经济发展。进一步完善、落实鼓励全民创业的政策和措施，加大对民营企业的扶持力度。保护和调动投资创业的积极性，引导民营企业加强管理，提升素质。加强和改进对民营企业的服务，创造公开公平公正的发展环境，让一切创造财富的源泉充分涌流。

　　(十)进一步优化发展环境，为完成各项工作任务提供保证

　　一是加快推进政府职能转变。强化市场监管，维护市场经济秩序。创新社会管理体制和管理方式，提高社会动态控制能力。进一步建立健全公共服务体系，提供更多、更满意的公共产品。加强公共行政服务中心建设，提高审批事项进中心率，解决体外循环问题，提高办事效率。二是大力推进依法行政。组织公务员特别是各级领导干部认真学习有关的法律法规，严格按照法定的权限和程序履行公务。自觉接受人大及其常委会的依法监督和人民政协的民主监督，认真执行人大及其常委会的各项决议。加强和改进人大代表建议、政协提案办理工作，推进办理工作尽快走上制度化、规范化、程序化轨道。加强民主政治建设，进一步拓宽与民主党派、工商联、人民团体及社会各界人士的联系渠道，建立民主决策机制，提高科学决策水平。深入贯彻《国务院全面推进依法行政实施纲要》，努力提高依法行政的能力和水平。加强制度建设，维护法制统一，确保政令畅通。落实行政执法责任制和过错追究制，强化法制监督，全面规范行政行为。认真搞好行政复议和仲裁工作，维护社会公平和正义。三是努力提高行政效能。坚持科学的发展观和正确的政绩观，建立和完善绩效考评体系，继续开展选优评差活动，做到令行禁止、奖惩分明，切实提高公信力和执行力。大力推进政务公开工作，继续办好行风热线和民心网，加快电子政务建设，提高工作透明度，降低行政运行成本。四是切实加强作风建设。多办顺应民意、化解民忧、为民谋利的实事。加大政务督查力度，推行问责制和责任追究制，对工作力度大、成效显著的，旗帜鲜明地表扬；对工作敷衍塞责、没有正当理由完不成任务的，予以严肃批评并追究责任。对决定了的重大事项要坚定不移，一抓到底，一气呵成，绝不半途而废。五是大力加强廉政建设。搞好廉洁自律，筑牢各级领导干部拒腐防变的思想堤坝。大力纠正部门和行业不正之风，认真受理域外投资客商的诉求，切实维护其合法权益，对破坏软环境的单位和个人严肃查处并公开曝光。以事关人民群众切身利益的问题为切入点，深入开展专项治理活动，加强源头防范工作，为全市经济和社会发展创造优良的政策环境、法制环境、服务环境和社会环境。

　　各位代表，过去的成就让我们站在了一个新的起点，人民的期待激励着我们实现新的跨越。让我们在市委的正确领导下，励精图治，乘胜奋进，为早日实现建设新铁岭大铁岭的宏伟目标而努力奋斗！
